# Bacterial communities in the rhizosphere, phyllosphere and endosphere of tomato plants

**DOI:** 10.1371/journal.pone.0223847

**Published:** 2019-11-08

**Authors:** Chun-Juan Dong, Ling-Ling Wang, Qian Li, Qing-Mao Shang

**Affiliations:** Institute of Vegetables and Flowers, Chinese Academy of Agricultural Sciences, Key Laboratory of Horticultural Crop Biology and Germplasm Innovation, Ministry of Agriculture, Beijing, P.R. China; Universite Paris-Sud, FRANCE

## Abstract

Plants harbor diverse bacterial communities, which play crucial roles in plant health and growth, in their rhizosphere, phyllosphere and endosphere. Tomato is an important model for studying plant-microbe interactions, but comparison of its associated bacterial community is still lacking. In this study, using Illumina sequencing of 16S rRNA amplicons, we characterized and compared the bacterial size and community from rootzone soil as well as the rhizosphere, phyllosphere and endosphere of roots, stems, leaves, fruits and seeds of tomato plants that were grown in greenhouse conditions. Habitat (soil, phyllospheric, and endophytic) structured the community. The bacterial communities from the soil-type samples (rootzone soil and rhizosphere) showed the highest richness and diversity. The lowest bacterial diversity occurred in the phyllospheric samples, while the lowest richness occurred in the endosphere. Among the endophytic samples, both bacterial diversity and richness varied in different tissues, with the highest values in roots. The most abundant phyla in the tomato-associated community was Proteobacteria, with the exception of the seeds and jelly, where both Proteobacteria and Firmicutes were dominant. At the genus level, the sequences of *Pseudomonas* and *Acinetobacter* were prevalent in the rhizosphere, and in the phyllosphere, more than 97% of the sequences were assigned to *Acinetobacter*. For the endophytes, *Acinetobacter*, *Enterobacter*, and *Pseudomonas* were the abundant genera in the roots, stems and leaves. In the fruits, the bacterial endophytes varied in different compartments, with *Enterobacter* being enriched in the pericarp and seeds, *Acinetobacter* in the placenta, and *Weissella* in the jelly. The present data provide a comprehensive description of the tomato-associated bacterial community and will be useful for better understanding plant-microbe interactions and selecting suitable bacterial taxa for tomato production.

## Introduction

Plants are colonized by complex bacterial communities that play different roles in plant growth and health [1]. Some bacteria are pathogenic and cause diseases, and others can promote plant growth by enhancing nutrient acquisition and tolerance to biotic and abiotic stresses, but a large fraction of these bacteria have no known function to their hosts. Depending on the plant environment, the bacteria associated with plants can be found on the exterior of plants, such as the rhizo- or phyllosphere, or in the interior of plants, referred to as the endosphere [2].

The rhizosphere is a hot spot for numerous organisms and is considered one of the most complex ecosystems on Earth [3]. Because of their high diversity and direct involvement in plant growth and diseases, rhizospheric bacteria, especially plant growth-promoting rhizobacteria (PGPR), have been extensively studied to elucidate their activities and functions. On the one hand, PGPR can directly promote plant growth via phytohormone production, nutrient solubilization, and nitrogen fixation and metabolism [4]. On the other hand, indirect effects of PGPR on plant growth promotion are related mainly to the suppression of soil-borne pathogenic and deleterious microorganisms by exclusion and antagonism [5]. Additionally, plant pathogenic bacteria can colonize the rhizosphere by striving to break through the protective microbial shield and to overcome the innate plant defense mechanisms in order to cause disease [5]. Numerous studies have clearly shown that the plant genotype and the soil type are two main drivers that shape the rhizosphere microbiome [5,6].

The phyllosphere is the microbial habitat defined by the surface of aboveground plant organs. The phyllosphere represents the largest microbial habitat on Earth, and bacteria are the most prevalent phyllosphere-colonizing microbes (also defined as epiphytes) [2,7]. In contrast to the comparatively weak and buffered fluctuations of environmental conditions in the rhizosphere, the phyllosphere is an extreme and unstable habitat. The bacterial epiphytes in the phyllosphere are exposed to acute fluctuations in temperature, humidity, and UV light irradiation and face limited access to nutrients [2,7,8]. In addition to environmental variability, epiphytes also encounter antimicrobial compounds that are produced by plants or other microorganisms [8].

Bacterial communities can also live and thrive inside their host plants, which are called endophytes [9]. Endophytic bacteria have been isolated from different parts of plants that are above and below ground including roots, stems, leaves, flowers, fruits, tubers, seeds and ovules [10]. Bacterial endophytes normally complete their life cycle within host plants without subjecting the plants to any disadvantages, but their multiplication might be limited by the innate immune system of host plants [11].

Most phyllospheric and endophytic bacteria act as commensals without any known effect on their plant host, but multiple bacteria establish a mutualistic relationship with plants. The host plants supply the bacteria with nutrients and shelter, and in turn, the bacteria can promote the growth of the host plants and offer resistance against insects, pests, and pathogens [11,12]. Certainly, some epiphytes and endophytes might be pathogenic. The outcome of plant-bacteria interactions depends on the environmental factors, the host genotypes, and the interacting microbes [1]. Illustrating the bacterial communities from the phyllosphere and endosphere is useful to uncover potentially beneficial candidates for biological control. In recent years, cultivation-independent approaches, especially metagenomics approaches, allow the full depth analysis of phyllospheric and endophytic bacteria diversity from various types of plant hosts, including agronomic crops, prairie plants, and naturally growing trees [9,13].

Tomato (*Solanum lycopersicum* L.) is widely grown and constitutes a major agricultural industry worldwide (http://faostat.fao.org). This species is also used as an excellent model for basic and applied research on fruit quality, plant-microbe interactions and other physiological traits [14,15]. Diseases are one of the main problems of the tomato industry worldwide, and biological control agents have emerged as an alternative approach for the control of tomato diseases [16,17]. Characterization of bacterial communities associated with tomato plants will contribute to not only exploring the mechanisms of selectivity in bacterial colonization in different compartments of plants but also identifying potential candidates for biologic control [18,19]. Recently, the diversity of bacteria associated with tomato has been studied in a few reports [20–24]. However, these studies focused on rhizospheric, phyllospheric or endophytic communities separately or analyzed the bacterial communities in a single tissue. A comprehensive analysis of the bacterial diversity of epiphytes and endophytes in different tissues and their correlation with soil bacteria is still lacking. Moreover, most of the previous literature has involved field-grown tomato plants [18,25]. Little information is available about the bacterial community structure of tomato cultivated in a greenhouse environment, which has been widely used in tomato production.

In this study, using Illumina-based 16S rRNA gene sequencing, we characterized and compared the bacterial community size and structure of the rhizosphere, phyllosphere and endosphere of roots, stems, leaves, fruits and seeds from tomato plants, which were grown in greenhouse conditions. This study provides comprehensive insight into the bacterial communities associated with tomato cultivated in a greenhouse agro-ecosystem and provides useful information for the control of potential pathogens in tomato cultivation.

## Materials and methods

### Collection of tomato plants

Tomato plants (*S*. *lycopersicum* cultivar “Zhongza 302”) were collected from the research greenhouse located at the Institute of Vegetables and Flowers, CAAS (116°20′21′′ E and 39°58′9′′ N). Seedlings were started in a glass-covered greenhouse on March 6, 2017, and then transplanted to a plastic-covered greenhouse on April 21, 2017. Plants were irrigated every two days according to water needs with drip tapes. Insect control, weed control and fertilization were accomplished following the recommendation of the seed company. Tomato plants were collected on July 14, 2017. All of the sampled plants were healthy-looking. For each sample, three biological replicates were prepared, and each replicate contained 20 plants.

Wearing sterile gloves, we collected three uninjured leaves from the bottom of selected plants. Two 5-cm stem cuttings between the second and fourth leaves were collected. Two or three red and mature fruits per plant were taken from various locations. Then, the plants were dug out with intact roots. By shaking roots vigorously, the root-zone soil was collected then sieved through a 2-mm sieve. All soil and plant samples were placed separately in ziplock bags, taken back to the lab and stored at 4°C until bacterial isolation.

### Isolation of rhizospheric, phyllospheric and endophytic bacteria

To recover the rhizospheric microbes, root samples were washed twice with sterilized PBST buffer (Na_2_HPO_4_ 1.42 g/L; KH_2_PO_4_ 0.24 g/L; NaCl 8 g/L; KCl 0.2 g/L; 0.01% Triton X-100, pH 7.4) with shaking (150 rpm) for 1 h at 30°C. After centrifugation at 1000 rpm at 4°C, the soil pellets were collected as rhizospheric samples and stored at -80°C prior to DNA extraction. The roots were continued to be washed until soil particles were completely removed. Then the washed roots were cut up in pieces and a subsample of root tissue, representative of whole root system, including young fresh roots as well as older root tissues, was collected and used for isolation of endophytic bacteria.

For the phyllospheric bacteria, the stem and leaf samples were washed twice with sterilized PBST buffer with shaking (150 rpm) for 1 h at 30°C. After centrifugation, the microbial pellets were collected as epiphytes and stored at -80°C.

Then, the washed root pieces, stems and leaves were disinfected by placing them for 2 min in 80% ethanol followed by 10 min in 5% sodium hypochlorite (NaClO, containing 0.01% Triton X-100) and then rinsed three times with sterilized distilled water. To validate our disinfection process, 100 μL of the third rinse was added to tryptic soy agar (TSA) plates, and no bacterial growth was observed after 3 days of incubation at 30°C. The disinfected samples were ground with a sterilized mortar and pestle and then incubated in PBS buffer for 2 h. The tissue incubations were filtered through four layers of gauze to remove the residuals. After centrifugation, the microbial pellets were harvested as endophytes.

For each part of the fruit samples, fruits were disinfected and washed, and then cut in half. The slurry containing the seeds were collected in a sterilized beaker, and the remainder were used to separate the pericarp and placenta. For the separation of jelly-like parenchyma and seeds, the slurry was rubbed in a sterilized gauze. The gauze containing jelly was then washed in the sterilized PBST buffer with shaking. After centrifugation, the microbial pellets were collected as the jelly endophytes. Then the separated seeds were washed under running tap water and surface-sterilized with NaClO solution. The collected seeds, pericarp and placenta samples were then grounded and incubated in the PBS buffer. The endophytic microbes from each part of fruits were isolated in a similar way with the leaf and stem tissues.

### DNA extraction, PCR amplification, and sample pooling

Altogether, there were 33 samples: two soil samples, two phyllospheric samples and seven endophytic samples, and three replicates were included for each sample. DNA was extracted using a FastDNA^®^ Spin Kit for Soil (MP Bio, Santa Ana, CA, USA) according to the manufacturer’s protocol. The extracted DNA was further purified with a TIANquick Midi Purification Kit (TIANGEN Biotech Co. Ltd., Beijing). DNA was quantified with an ND 1000 spectrophotometer (NanoDrop, Thermo Scientific, Wilmington, DE) and adjusted to a final concentration of 2.5 ng/μL. The DNA integrity was further confirmed by 0.8% agarose gel electrophoresis.

Primers 338F (5’-ACTCCTACGGGAGGCAGCAG-3’) and 806R (5’-GGACTACHVGGGTWTCTAAT-3’) were used to amplify V3 and V4 of the 16S rRNA gene [26]. Each 20 μL PCR contained 10 ng of DNA, 250 μM dNTPs, 200 nM forward primer, 200 nM reverse primer, 12.5 μg ultrapure BSA (Ambion), FastPfu Buffer, and 1 unit of TransStart FastPfu DNA Polymerase (TransGen). Cycling conditions were 94°C for 3 min, followed by 25 cycles of 94°C for 30 sec, 55°C for 30 sec, and 72°C for 45 sec, with a final extension of 72°C for 10 min. All samples were amplified in quadruplicates, which were combined before purification. PCR products were separated on a 2% agarose gel, and the bacterial products were extracted from the gel using the AxyPrep^™^ DNA Gel Extraction Kit (Axygen, USA). DNA was quantified with a QuantiFluor^®^ dsDNA System (Promega, Madison, WI, USA), and the quality was checked using an Agilent Bioanalyzer 2100 system. The DNA concentration was adjusted to 1 ng/μL. The amplicon libraries were prepared by pooling 10 ng of each PCR. Finally, the libraries were sequenced on the HiSeq2500 platform (Illumina, CA, USA) with the generation of 2 × 250 base pairs (PE250) at Shanghai Majorbio Bio-Pharm Technology Co., Ltd.

### Sequence analysis

The raw Fastq reads were processed using a custom pipeline developed at the Joint Genome Institute [27,28]. The software package Mothur (version 1.31.2) was used for sequence analysis, following the Standard Operating Procedure as described previously [29]. Chimeric sequences were detected with UCHIME [30] and subsequently removed from the dataset. Next, sequences were quality trimmed, merged and clustered using the furthest neighbor clustering algorithm to build OTUs (operational taxonomic units). The resulting file was parsed to separate the data for each sample. OTUs were assigned a taxonomic group with classify.seqs using the RDP reference file and a cutoff of 80% of the bootstrap value. For description of the community, OTUs with the same taxonomy were binned together at the phylum, class and genus levels. Sequences matching “Cyanobacteria_Chloroplast” and “Mitochondria” were removed [31]. The sequencing data have been uploaded in the supplemental material (S1 File), and the raw data have been uploaded to NCBI Sequence Read Archive (SRA, PRJNA576345).

### Statistical analysis

All samples were normalized at the same sequence depth. Rarefaction curves and Shannon-Wiener curves were generated to estimate the sequencing depth and to compare the relative levels of bacterial richness among different samples [32]. OTUs were used to calculate α-diversity indices (Chao1 and Shannon) using in-house Perl Scripts [33]. For β-diversity analysis, Principal Coordinates Analysis (PCoA) and Bray-Curtis dissimilarity index were used to study community structures across all samples [34]. PCoA was performed based on weighted and unweighted UniFrac distance matrices, and a two-dimensional plane was used to determine whether communities with similar characteristics tend to cluster together. The statistical significance of the differences in three sample groups (soil, phyllospheric, and endophytic) was assessed through Analysis of Similarity (ANOSIM) testing [35]. Two-way analysis of variance (ANOVA) was used to test the effects of “habitat” (soil, phyllospheric, and endophytic) on the relative abundance of bacterial members [31]. ANOVA analyses were performed with SPSS for Windows statistical software (SPSS Inc., Chicago, IL). Paired student’s *t*-tests were calculated for all pairwise comparisons, and *P* values were adjusted using the FDR correction for multiple testing.

## Results

### Analysis of sequencing data

Illumina sequencing of bacterial *16S rRNA* genes yielded 1,323,922 valid sequences. After quality trimming, 1,228,449 trimmed sequences were obtained (S1 Table). We found that primer pair 338F/806R could amplify both bacterial and plant chloroplast DNA under our PCR conditions. The proportion of reads assigned to a plant taxonomic identification ranged from 0 to 68% for each sample. After removing reads assigned to the taxonomic kingdom Plantae, 847,914 sequences remained. These sequences were clustered into 1,443 OTUs with 97% similarity. To compare samples, the number of sequences per sample was standardized to the minimum number of sequences in a single sample (16,966 sequences), and a total of 1,374 OTUs were obtained (S1 Table).

**Fig 1:**
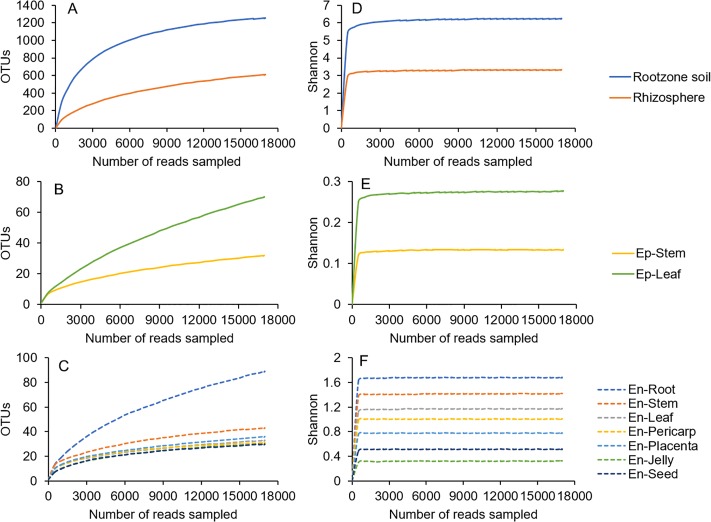
Rarefaction curves of soil (A, D), phyllospheric (B, E) and endophytic (C, F) bacteria based on OTU numbers (A-C) and Shannon indices (D-F).

At this sequencing depth, both rarefaction curves and Shannon-Wiener curves began to level off, suggesting that the plant-associated communities were reasonably well characterized with our sampling effort (Fig 1). Interestingly, the curves of soil samples were much higher than the phyllospheric and endophytic samples.

### Richness and diversity of bacterial communities

**Fig 2:**
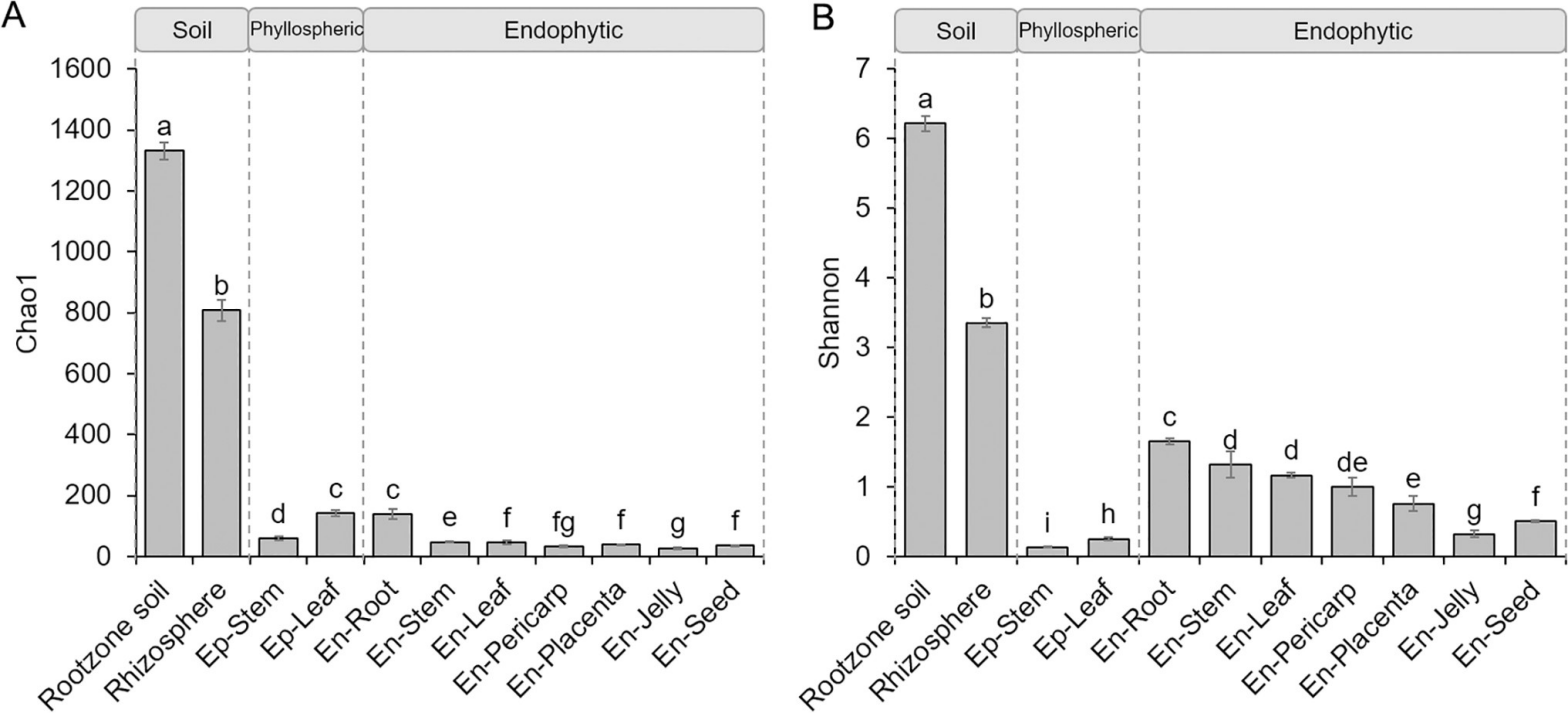
Chao1 (A) and Shannon (B) indices of bacterial communities from the root zone soil, rhizosphere, phyllosphere and endosphere of tomato plants.

The Chao index was applied to measure the richness of the bacterial communities (Fig 2A). The bacteria in the root zone soil showed the highest richness (1347) among all of the tested samples, followed by the bacteria in the rhizosphere (920). For the phyllospheric bacteria, the richness was higher in the leaves (Ep-Leaf) than in the stems (Ep-Stem), while the reverse was true for the endophytic samples. The endophytic bacterial richness in the roots was identical to that in the Ep-Leaf sample and much higher than that in other endophytes from other tissues.

The Shannon index was further analyzed to represent the diversity of bacterial species (Fig 2B). Similar to the richness analysis, the soil bacteria showed the highest diversity, and the bacteria from the root zone soil were more diverse than those from the rhizosphere. For the phyllospheric samples, the bacteria from the Ep-Leaf sample were more diverse than those from the Ep-Stem sample. However, in contrast to the richness indices, the diversity of endophytic bacteria was higher than that of the epiphytes. Among the different tomato tissues, the bacterial diversity decreased in the following order: roots > stems ≈ leaves > pericarp > placenta > seeds > jelly (Fig 2B). The richness and diversity indices also showed a similar pattern for the sequence data before subsampling (S1 Fig).

### Bacterial communities differentiated by habitat type

**Fig 3:**
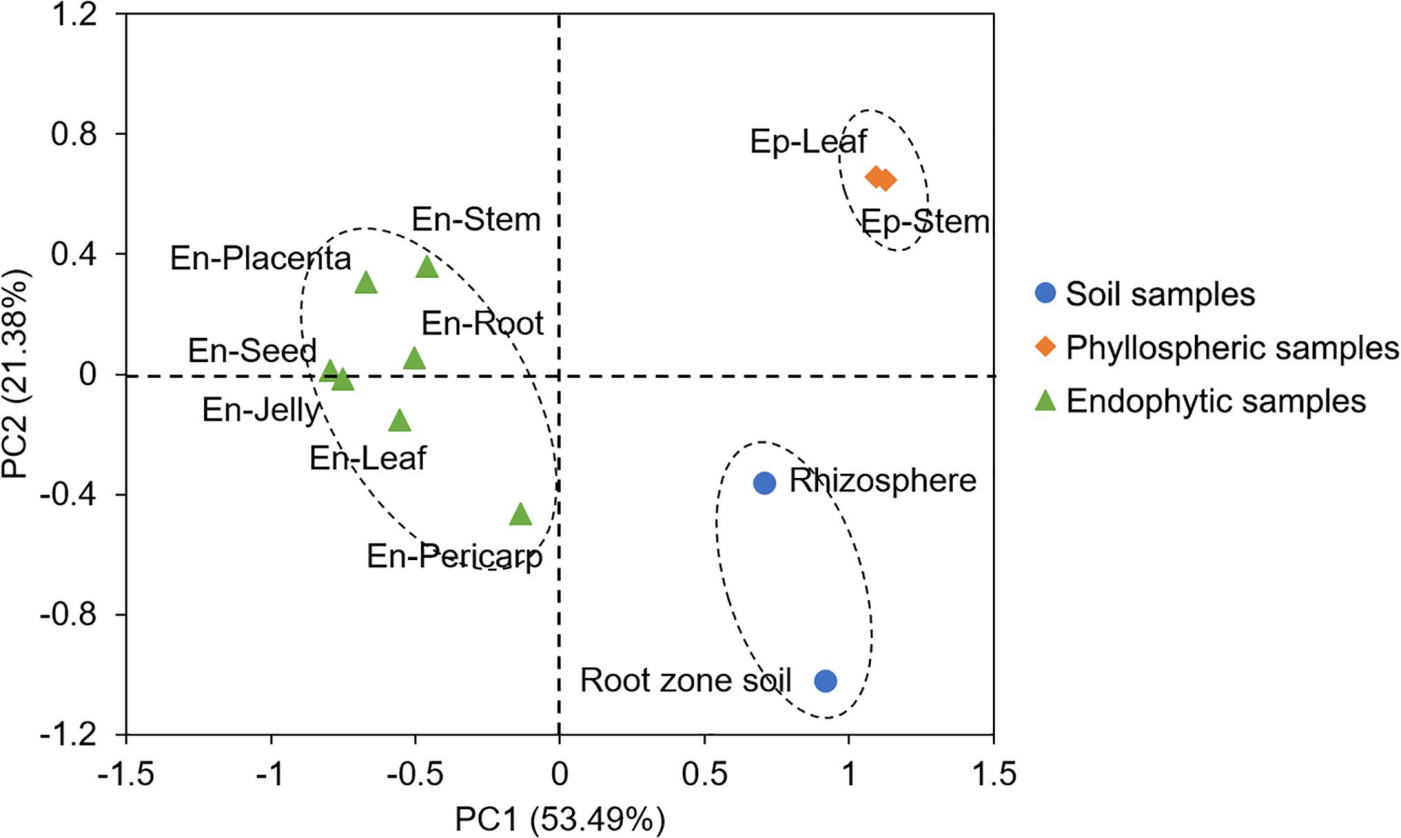
PCoA analysis of bacterial communities of soil, phyllospheric and endophytic samples. **PC1 represents the maximum variation factor, and PC2 represents the second principal coordinate.** Samples that cluster close together share a greater similarity in composition.

PCoA analysis was performed to test the effect of ‘habitat’ (soil, phyllospheric, and endophytic) on the bacterial compositions. The results showed that there were significant differences in the bacterial communities among the three sample groups with different habitats. Samples in each group were clustered together into their own area (Fig 3). PC1 and PC2 accounted for 53.49% and 21.38% of the total changes, respectively. Additionally, in the phyllospheric group, the stem and leaf epiphytes clustered closely together, indicating that the bacterial communities were very similar. Similarly, in the endophytic groups, the communities of En-Jelly and En-Seed were also clustered closely. However, the endophyte communities from the pericarp were relatively distinct from the others, which might be correlated with the additional presence of *Rosenbergiella nectarea* (OTU397, Table 1). The similar results were also identified in the Bray-Curtis dissimilarity analysis (S2 Fig). ANOSIM testing confirmed the significant differences in the microbial composition among the same ‘habitat’ grouping at PCoA. There was a significant difference either between soil and phyllospheric samples (R^2^ = 0.01, *P* = 0.021), or between phyllospheric and endophytic samples (R^2^ = 0.008, *P* = 0.0093).

**Table 1:** The bacterial communities enriched in the rhizosphere, phyllosphere and endosphere of tomato plants.

OTU ID	Species (or higher)	Phylum	Relative abundance (%)											ANOVA test	
			Soil		Phyllospheric		Endospheric							Habitat	
			Root zone soil	Rhizosphere	Ep-Stem	Ep-Leaf	En-Root	En-Stem	En-Leaf	En-Pericarp	En-Placenta	En-Jelly	En-Seed	F	*P*
OTU62	OTU62	Cloacimonetes	n.d.	n.d.	n.d.	n.d.	n.d.	n.d.	n.d.	n.d.	n.d.	n.d.	3.94	0.242	0.790
OTU120	*Weissella cibaria*	Firmicutes	n.d.	n.d.	n.d.	0.01	n.d.	n.d.	0.003	1.16	0.08	70.60	n.d.	0.252	0.783
OTU200	*Acinetobacter*	Proteobacteria	0.04	17.38	98.37	97.11	27.27	63.29	8.19	0.03	90.56	8.22	1.73	5.016	0.039
OTU383	*Enterobacter*	Proteobacteria	0.06	3.26	0.71	1.83	42.59	17.29	75.40	83.20	6.97	13.33	61.42	7.000	0.030
OTU397	*Rosenbergiella nectarea*	Proteobacteria	n.d.	n.d.	0.012	0.016	n.d.	0.004	0.09	14.42	0.59	1.05	0.16	0.321	0.735
OTU502	*Rhizobium giardinii*	Proteobacteria	0.085	2.08	n.d.	n.d.	0.012	n.d.	n.d.	n.d.	n.d.	n.d.	n.d.	7.374	0.025
OTU628	*Bacteroides thetaiotaomicron*	Bacteroidetes	0.01	0.01	0.05	0.05	0.01	0.15	0.09	0.04	0.11	2.21	4.25	0.565	0.589
OTU663	*Pantoea*	Proteobacteria	n.d.	0.004	0.38	0.07	0.06	7.84	0.12	0.71	0.01	n.d.	n.d.	0.260	0.777
OTU786	*Acinetobacter*	Proteobacteria	0.002	1.22	n.d.	0.04	2.38	n.d.	0.07	n.d.	0.01	n.d.	n.d.	0.254	0.782
OTU872	*Rhizobium* sp. IRBG74	Proteobacteria	0.04	4.71	n.d.	n.d.	0.04	n.d.	n.d.	n.d.	n.d.	n.d.	n.d.	6.659	0.036
OTU918	*Pseudomonas*	Proteobacteria	0.20	16.09	0.04	0.04	10.27	10.54	13.67	n.d.	0.08	0.05	0.47	3.431	0.180
OTU970	*Staphylococcus*	Firmicutes	0.002	n.d.	n.d.	0.002	0.02	0.03	n.d.	0.005	0.035	0.16	1.26	0.364	0.706
OTU1110	*Lachnospiraceae* NK4A136 group	Firmicutes	n.d.	0.002	n.d.	0.004	n.d.	n.d.	0.01	0.003	0.02	0.42	2.21	0.361	0.708
OTU1117	Bacteroidales S24–7 group	Bacteroidetes	n.d.	n.d.	n.d.	n.d.	n.d.	n.d.	n.d.	n.d.	0.01	0.05	1.89	0.261	0.777
OTU1194	*Pseudomonas*	Proteobacteria	0.01	2.01	n.d.	n.d.	0.17	0.01	0.01	0.003	0.01	n.d.	n.d.	4.566	0.102
OTU1274	*Ensifer*	Proteobacteria	0.19	7.13	n.d.	n.d.	0.03	n.d.	n.d.	n.d.	n.d.	n.d.	n.d.	7.374	0.025
OTU1376	*Pseudomonas brassicacearum* subsp. brassicacearum	Proteobacteria	0.10	16.91	n.d.	n.d.	14.36	0.004	n.d.	n.d.	n.d.	n.d.	n.d.	1.065	0.389
OTU1387	*Clostridiales bacterium* CIEAF020	Firmicutes	n.d.	0.002	0.01	0.03	n.d.	0.03	0.03	0.01	0.03	0.47	3.31	0.334	0.726

n.d., not detected.

Furthermore, ANOVA was used to test the effect of ‘habitat’ on the relative abundance of bacterial communities. For the two common OTUs identified in all samples, OTU200 and OTU383, a significant effect of habitat was found (*P* < 0.05). A significant effect was also found for the other two OTUs, OTU502 and OTU782, which were present abundantly in only soil samples (Table 1).

### Taxonomic distributions of rhizospheric, phyllospheric and endophytic bacteria

**Fig 4:**
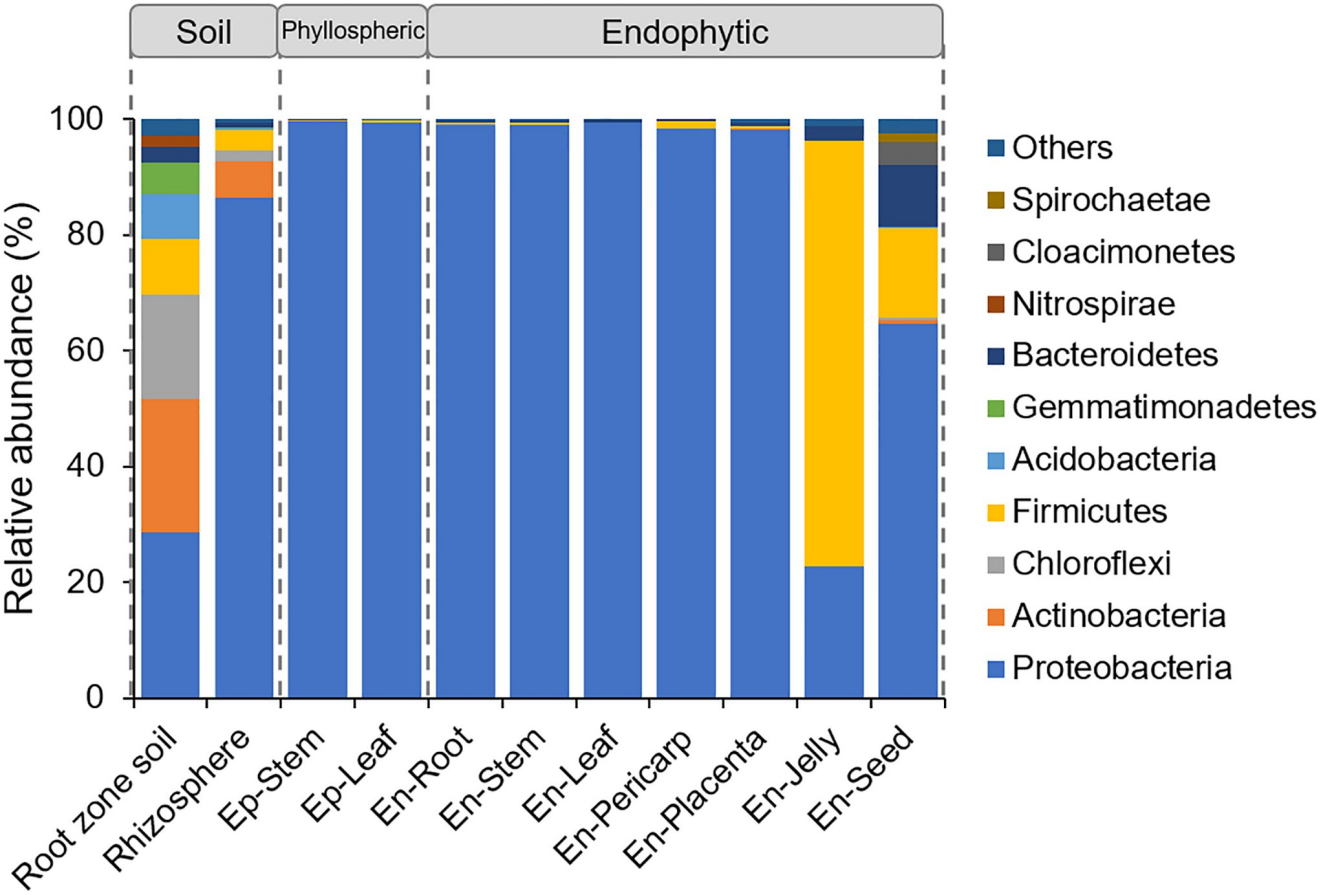
Distribution of the bacteria from the root zone soil, rhizosphere, phyllosphere and endosphere of tomato plants at the phylum level.

First, the taxonomy of the sequences was examined at the phylum level on the basis of the RDP Bayesian Classifier (Fig 4). In the soil from the root zone, the heavily sequenced phyla included Proteobacteria (28.72%), Actinobacteria (22.98%), Chloroflexi (17.91%), Firmicutes (9.71%), Acidobacteria (7.74%), and Gemmatimonadetes (5.45%). However, only the Proteobacteria phylum was enriched in the rhizospheric, phyllospheric and endophytic samples, with the exception of the seed and jelly samples, of which both Proteobacteria and Firmicutes were the dominant endophytes. In the seeds, sequences assigned to the Bacteroidetes, Cloacimonetes and Spirochaetae were additionally enriched (Fig 4).

**Fig 5:**
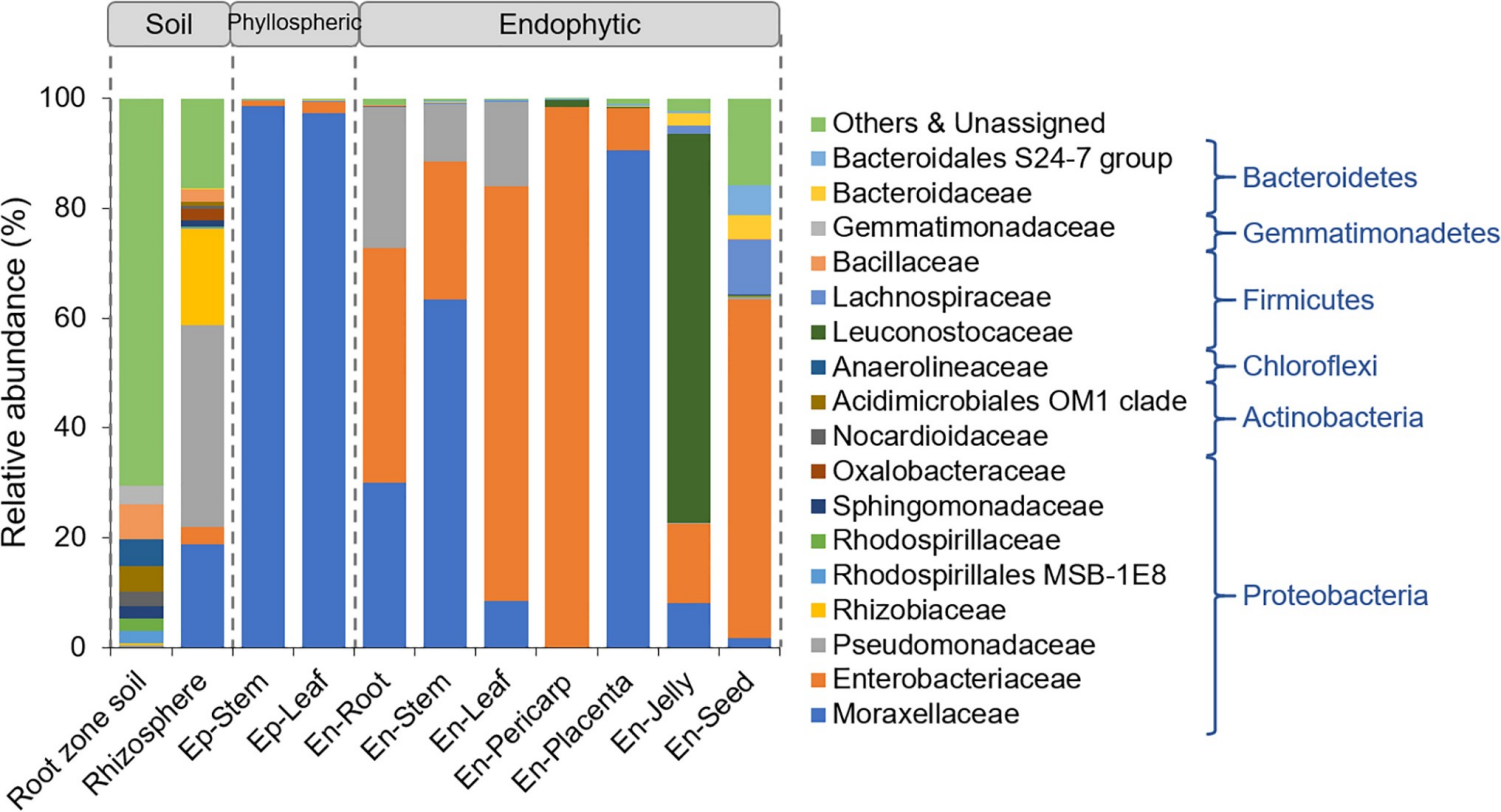
Distribution of the bacteria from the root zone soil, rhizosphere, phyllosphere and endosphere of tomato plants at the family level.

At the family level, the Proteobacteria bacteria in the rhizosphere were mainly assigned to the families of Moraxellaceae, Pseudomonadaceae and Rhizobiaceae, while for the phyllospheric bacteria, 98.52% and 97.34% of sequences were assigned to the Moraxellaceae family in the Ep-Stem and Ep-Leaf samples, respectively (Fig 5). For the endophytic bacteria in the roots, stems and leaves, the Proteobacteria bacteria included three families: Moraxellaceae, Enterobacteriaceae and Pseudomonadaceae. These three families were almost evenly distributed in the root, while in the stems and leaves, Moraxellaceae and Enterobacteriaceae were the most abundant families, respectively. In the fruits, the Proteobacteria bacteria were mostly assigned to the family Enterobacteriaceae in the pericarp, and Moraxellaceae was additionally present in the placenta (Fig 5). For the endophytic bacteria from the jelly around the seeds, 73.61% of sequences were assigned to the Firmicutes phylum, which mainly included the Leuconostocaceae family, and 22.66% of sequences were assigned to the Proteobacteria phylum, which was composed of the families of Enterobacteriaceae (14.38%) and Moraxellaceae (8.22%). In the seeds, the Firmicutes bacteria were mainly annotated to the family of Lachnospiraceae, and the Bacteroidetes phylum consisted of Bacteroidaceae and the S24–7 group.

**Fig 6:**
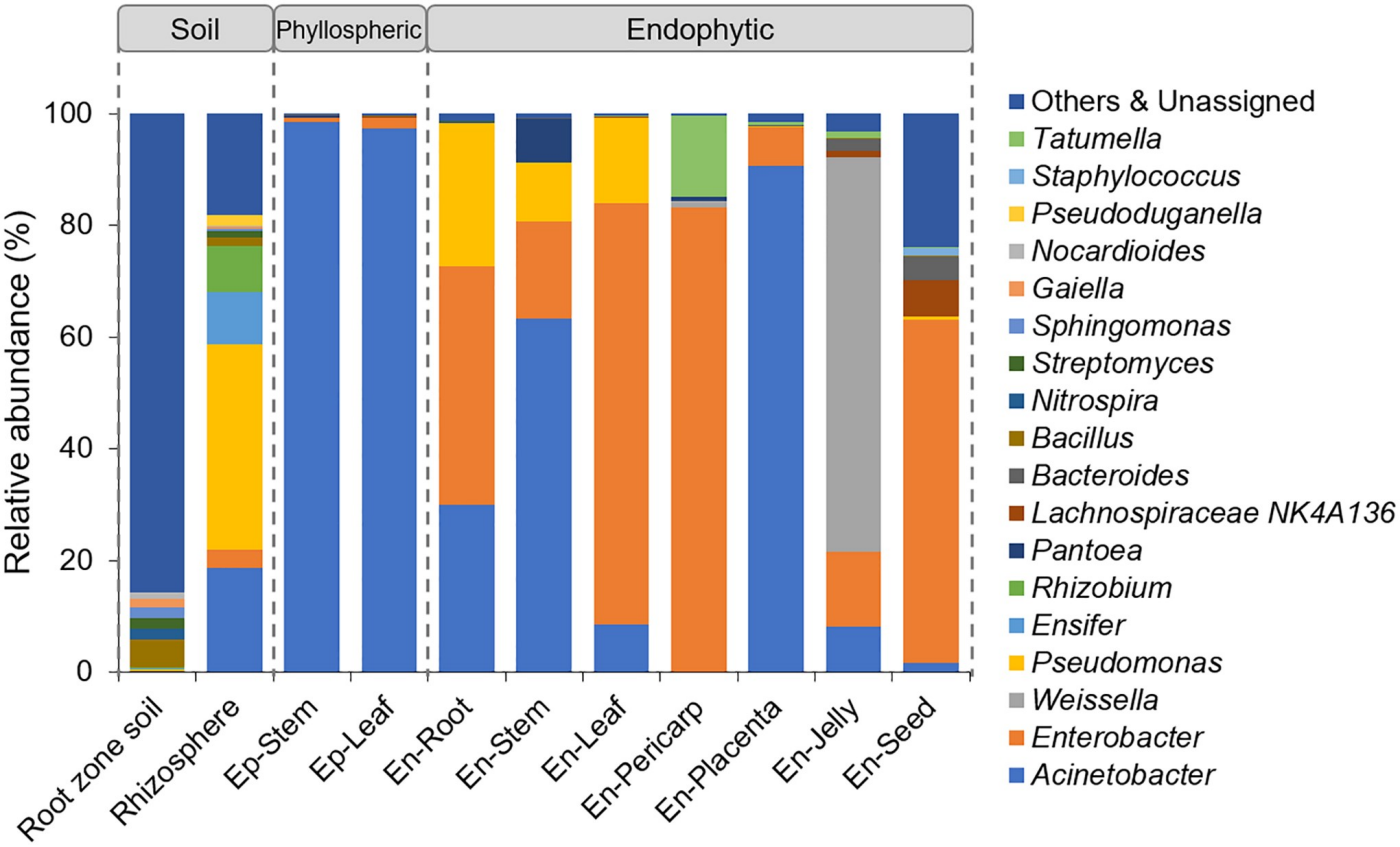
Distribution of the bacteria from the root zone soil, rhizosphere, phyllosphere and endosphere of tomato plants at the genus level.

At the genus level, only approximately 37% of the sequence reads could be classified into 248 known genera, and most of them were only present in soil samples. Here, the top 18 genera with relative abundances greater than 1% in at least one sample were used to compare the genus compositions among different samples (Fig 6). Although *Bacillus* was the most abundant genus in the root zone soil (4.99%), it only accounted for 1.53% of the rhizospheric bacteria. In the rhizosphere, some genera were selectively enriched. Of these genera, *Pseudomonas* and *Acinetobacter* were most enriched, accounting for 36.76% and 18.72%, respectively, followed by *Ensifer* (9.30%) and *Rhizobium* (8.16%). For the phyllospheric bacteria, the genera composition was very simple, with more than 97% of sequences being assigned to *Acinetobacter*. For the endophytes, in the roots and leaves, *Acinetobacter*, *Enterobacter*, and *Pseudomonas* accounted for more than 98% of the sequences, and in the stems, *Pantoea* was additionally present with a relative abundance of 7.84%. Compared to the vegetative tissues, the compositions of bacterial endophytes in the fruits were quite different, and their distributions also varied in different compartments. In the pericarp, *Enterobacter* was the most abundant genus (83.20%), followed by *Tatumella* (14.42%). In the placenta, *Acinetobacter* and *Enterobacter* accounted for 97.54% of the total bacteria. However, in the jelly on the surface of the seeds, the most predominant genus was *Weissella* (70.60%), and *Acinetobacter* and *Enterobacter* accounted for only 13.33% and 8.22% of the total sequences, respectively. Although the seeds were surrounded by the jelly, the bacterial genera were different between them. In the seeds, 61.42% of sequences were clustered as the genus *Enterobacter*. Two genera, *Lachnospiraceae NK4A136* and *Bacteroides*, were additionally present in the seeds, with relative abundances of 6.46% and 4.25%, respectively.

We noticed that predominance at the genus level was driven by the high abundance of one or two OTUs (Table 1). For example, *Acinetobacter* and *Enterobacter* were dominant in all rhizospheric, phyllospheric and endophytic communities due to the large number of OTU200 and OTU383. The genera *Pseudomonas* was mainly represented by sequences belonging to two OTUs, OTU918 and OTU1376. In the pericarp, *Tatumella* was represented by *Rosenbergiella nectarea* (OTU397). Similarly, *Weissella*, the most dominant in the jelly endophytic community, was represented by OTU120, which was assigned as *Weissella cibaria*.

## Discussion

Plants host distinct bacterial communities in the rhizosphere, phyllosphere, endosphere and surrounding soils, and these bacteria play a crucial role in plant growth and health [2,9]. Tomato is an important crop that is grown worldwide and is an excellent model for studying plant-microbe interactions [18]. The characterization of bacterial communities colonizing tomato is therefore valuable. In the present study, by Illumina amplicon sequencing, we examined and compared the bacterial communities in the rhizosphere, phyllosphere, endosphere, and root zone soil samples from greenhouse-cultivated tomato plants.

Based on OTU analysis and Chao1 and Shannon’s diversity indices, the bacterial richness and diversity from different habitats were compared. In general, the richness decreased from root zone soil to rhizosphere to phyllosphere to endosphere, while the diversity decreased in an altered order: root zone soil > rhizosphere > endosphere > phyllosphere (Fig 1). The lower richness and diversity in the phyllosphere samples compared to the soil samples are understandable. And this finding was consistent with the previous reports in other plant species, including Arabidopsis, soybean, rice, Agave and some of its relatives s [36, 37]. Intuitively, root and leaf tissues will have different total bacterial population sizes. In the phyllosphere, the bacterial abundance is estimated to be 10^7^ cells/cm^2^ or approximately 10^6^ cells/g, while the bacterial number in the rhizosphere may reach up to 10^8^ cells/g dry weight root tissue [38–40]. The bacterial diversity was positively correlated with the total community size.

The richness of endophytes in the stems and leaves was lower than that in the phyllospheric communities. However, the reverse pattern was found for the bacterial diversity (Fig 2). The source of bacterial inoculum might be a major reason. The bacteria in the phyllosphere come from either soil, wind, air or water, which are enriched with microbes [41], while only some phyllospheric bacteria would be selected to enter the leaves and stems [7]. This might explain the higher richness in the phyllosphere compared to the endophytes. However, phyllospheric bacteria are directly exposed to high UV radiation, higher temperature gradients and antimicrobial pesticides, and only bacteria with high resistance can survive in the phyllosphere [2,42]. Over time, the endophytic bacteria from the roots could also migrate or be transported to the above-ground parts of plants [36]. All of these could explain the lower diversity in the phyllosphere compared to the endophytic community from the stems and leaves. In this study, the relatively closed environment provided by the greenhouse might also be another reason that the diversity of phyllospheric bacteria was limited. Moreover, the bacterial richness and diversity of the endophytes varied in different tissues, with the highest bacterial diversity occurring in roots. Similar results were also reported for other plant hosts, such as *Arabidopsis* [31], rice [43], and *Agave* species [28]. Most root endophytic bacteria are from soil, which represents one of the richest microbial ecosystems on Earth [44]. The highest endophytic bacterial diversity observed in the root samples may be attributed to the primary site of interaction between plants and soil [45,46]. Furthermore, an interesting gradient was observed with regard to the distance of each plant part from the soil: the diversity of bacterial endophytes decreased as the distance from soil increased, similar to the finding for the epiphytes of tomato plants [21].

Comparison of the bacterial communities associated with tomato plants reveals both ubiquitous and specific members in different sample types. Proteobacteria, Actinobacteria, Chloroflexi, Firmicutes, Acidobacteria and Gemmatimonadetes were the abundant phyla in the root zone soil, while in the rhizosphere, only Proteobacteria were enriched, and of this phylum, *Pseudomonas*, *Acinetobacter*, *Enterobacter* and *Rhizobium* were the abundant genera (Figs 3 and 4), confirming the results from other studies on the tomato rhizosphere [21,47]. The genera *Pseudomonas*, *Acinetobacter*, and *Enterobacter* could further colonize roots. However, some bacterial genera identified in the tomato roots at the fruiting stage grown in the fields, such as *Chryseobacterium*, *Leifsonia*, *Pandoraea*, *Dokdonella*, and *Arthrobacter* [21], were not detected in our study. This might be due to the indoor environment in greenhouses, which limited the total microbial diversity. Certainly, the differences in tomato cultivars and climatic conditions might be the other reasons for the different endophytic compositions. Additionally, most of these root endophytes have potentially beneficial effects on plant growth and health. For example, *Pseudomonas brassicacearum* subsp. *brassicacearum* (OTU1376) presented high ACC deaminase activity, which could hydrolyze ACC and thereby decrease ethylene levels and promote plant growth [48].

In this study, in the phyllosphere of tomato plants, only *Acinetobacter* was abundant (more than 97% of sequences), and the epiphytic bacterial communities from stems and leaves showed high similarity, but this bacterial distribution was quite different from the previous report of tomato plants, which was carried out in other locations. Ottesen et al. [21] found that epiphytes of bottom leaves and stems of tomato plants were mainly composed of *Xanthomonas*, *Rhizobium*, *Methylobacterium*, *Sphingomonas*, and *Pseudomonas*. All of these were absent in our phyllosphere samples. Furthermore, compared to the other host plants, we did not detect many sequences for *Bacillus* and *Pantoea*, which dominate in the lettuce phyllosphere [49], or *Dyadobacter*, *Devosia* and *Pedobacter*, which are abundant in the potato phyllosphere [50]. The bacterial communities in the spinach phyllosphere were largely composed of *Pseudomonas* [51], but this genus was also rare in our samples. All of these findings support the conclusion that both geographical location and host species play important determinant roles in phyllospheric community composition [39].

Unlike the bacterial communities in the phyllosphere, the endophytes were more diverse in the tomato plants. In the vegetative tissues (roots, stems and leaves), the bacterial genera *Acinetobacter*, *Enterobacter*, *Pseudomonas* and *Pantoea* were abundantly present in these samples, albeit in different amounts (Fig 4). Of them, only *Acinetobacter* overlapped with a previous report performed on the tomato leaf endosphere [22], while the genera *Enterobacter*, *Pseudomonas* and *Pantoea* were also identified as endophytes in other plant hosts [9,10]. More specifically, the genera in roots, stems and leaves of the tomato plants were colonized by many of the same bacterial species (OTU200, OTU383, and OTU918), suggesting that many of the bacterial taxa in these tissues may come from similar sources. In this study, the stems and leaves were sampled at the bottom of tomato plants, and bacteria from leaves and stems may come from soil. In fact, these common bacterial endophytes were also observed abundantly in the soil samples (Table 1). A similar finding was also reported in *Arabidopsis*, whose leaves are close to the ground [31]. Another explanation for this finding is that the seeds are colonized from the soil, and as the plant grows, bacteria colonize the expanding roots, stems and leaves [52].

In tomato fruits and seeds, *Acinetobacter* and *Enterobacter* were shared as the common endophytes, but some new bacteria were additionally enriched. Colonization by these bacterial species might be attributed to the special internal environment in fruits, which are full of sugars and organic acids [53]. For example, Amari et al. [54] characterized a novel dextransucrase from *Weissella cibaria*. This enzyme catalyzes the synthesis of linear dextrans from sucrose and has broad applications in food industries. In tomato, *W*. *cibaria* (OTU120) was identified as the most dominant bacterial species in the jelly around the seeds (70.6% of sequences), indicating its potential function for the hydrocolloid-like properties of jelly. *Bacteroides thetaiotaomicron* (OTU628) was also enriched both inside and outside of tomato seeds. *B*. *thetaiotaomicron* is well known as an abundant commensal of the human gut and can digest a broad array of complex carbohydrates ranging from host glycans to plant cell wall pectins [55]. *Rosenbergiella nectarea* (OTU397) was abundant in the pericarp (14% of sequences). *R*. *nectarea* has been isolated from the flower nectar of *Amygdalus communis* (almond) and *Citrus paradisi* (grapefruit), which are also full of soluble sugars [56]. The presence of these bacteria would facilitate carbohydrate degradation during the development and ripening of tomato fruits and seeds. The substrate preference might be one of the explanations for the differentiation of endophytes in different fruit parts. In rhizosphere, the radical exudates and volatile compounds produced by plant roots exert strong selective forces in shaping bacterial assemblies [2,4]. Here, our results also suggested that the metabolic differences in different tissues might shape the endophytic bacterial compositions. Certainly, further experimental evidence is necessary.

In summary, the present study provides a holistic perspective of the composition, diversity and influential factors shaping the rhizospheric, phyllospheric and endophytic bacterial communities associated with greenhouse-grown tomato plants. Some potentially beneficial bacterial strains have been isolated in our laboratories, and their exact functions in tomato growth and health will be studied in the near future. These efforts will provide an important data resource for further application of the beneficial bacteria in tomato production.

## Supporting information

S1 File The fasta sequence, read amount, and IDs list for each OTU collected from the Illumina sequencing of the root zone soil, rhizospheric, phyllospheric and endospheric bacterial samples. (ZIP)

S1 Table Statistics of Illumina sequencing data of the root zone soil, rhizospheric, phyllospheric and endospheric bacteria associated with tomato plants. (DOCX)

S1 Fig Chao1 (A) and Shannon (B) indices of bacterial communities from the root zone soil, rhizosphere, phyllosphere and endosphere of tomato plants before subsampling. (DOCX)

S2 Fig Bray-Curtis dissimilarities across the bacterial communities from the root zone soil, rhizosphere, phyllosphere and endosphere of tomato plants. (DOCX)

## References

[1] Brader G, Compant S, Vescio K, Mitter B, Trognitz F, Ma LJ. et al Ecology and genomic insights into plant-pathogenic and plant-nonpathogenic endophytes. Annu Rev Phytopathol. 2017; 55: 61–83. 10.1146/annurev-phyto-080516-035641 28489497

[2] Bulgarelli D, Schlaeppi K, Spaepen S, Ver Loren van Themaat E, Schulze-Lefert P. Structure and functions of the bacterial microbiota of plants. Annu Rev Plant Biol. 2013; 64: 807–838. 10.1146/annurev-arplant-050312-120106 23373698

[3] Raaijmakers JM, Paulitz TC, Steinberg C, Alabouvette C, Moënne-Loccoz Y. The rhizosphere: a playground and battlefield for soilborne pathogens and beneficial microorganisms. Plant Soil 2009; 321: 341–361.

[4] Backer R, Rokem JS, Ilangumaran G, Lamont J, Praslickova D, Ricci E. et al Plant growth-promoting rhizobacteria: context, mechanisms of action, and roadmap to commercialization of biostimulants for sustainable agriculture. Front Plant Sci. 2018; 9: 1473 10.3389/fpls.2018.01473 30405652 PMC6206271

[5] Mendes R, Garbeva P, Raaijmakers JM. The rhizosphere microbiome: significance of plant beneficial, plant pathogenic, and human pathogenic microorganisms. FEMS Microbiol Rev. 2013; 37: 634–663. 10.1111/1574-6976.12028 23790204

[6] Bakker MG, Manter DK, Sheflin AM, Weir TL, Vivanco JM. Harnessing the rhizosphere microbiome through plant breeding and agricultural management. Plant Soil 2012; 360: 1–13.

[7] Vorholt JA. Microbial life in the phyllosphere. Nat Rev Microbiol. 2012; 10: 828–840. 10.1038/nrmicro2910 23154261

[8] Bringel F, Couée I. Pivotal roles of phyllosphere microorganisms at the interface between plant functioning and atmospheric trace gas dynamics. Front Microbiol. 2015; 6: 486 10.3389/fmicb.2015.00486 26052316 PMC4440916

[9] Afzal I, Shinwari ZK, Sikandar S, Shahzad S. Plant beneficial endophytic bacteria: Mechanisms, diversity, host range and genetic determinants. Microbiol Res. 2019; 221: 36–49. 10.1016/j.micres.2019.02.001 30825940

[10] Rosenblueth M, Martínez-Romero E. Bacterial endophytes and their interactions with hosts. Mol Plant Microbe Interact. 2006; 19: 827–837. 10.1094/MPMI-19-0827 16903349

[11] Singh R, Dubey AK. Diversity and applications of endophytic Actinobacteria of plants in special and other ecological niches. Front Microbiol. 2018; 9: 1767 10.3389/fmicb.2018.01767 30135681 PMC6092505

[12] Chaturvedi H, Singh V, Gupta G. Potential of bacterial endophytes as plant growth promoting factors. J Plant Pathol Microbiol. 2016; 7: 2.

[13] Müller T, Ruppel S. Progress in cultivation-independent phyllosphere microbiology. FEMS Microbiol Ecol. 2014; 87: 2–17. 10.1111/1574-6941.12198 24003903 PMC3906827

[14] Bai Y, Kissoudis C, Yan Z, Visser RGF, van der Linden G. Plant behaviour under combined stress: tomato responses to combined salinity and pathogen stress. Plant J. 2018; 93: 781–793. 10.1111/tpj.13800 29237240

[15] Karlova R, Chapman N, David K, Angenent GC, Seymour GB, de Maagd RA. Transcriptional control of fleshy fruit development and ripening. J Exp Bot. 2014; 65: 4527–4541. 10.1093/jxb/eru316 25080453

[16] Singh VK, Singh AK, Kumar A. Disease management of tomato through PGPB: current trends and future perspective. 3 Biotech. 2017; 7: 255 10.1007/s13205-017-0896-1 28730550 PMC5519495

[17] Raza W, Ling N, Zhang R, Huang Q, Xu Y, Shen Q. Success evaluation of the biological control of *Fusarium* wilts of cucumber, banana, and tomato since 2000 and future research strategies. Crit Rev Biotechnol. 2017; 37: 202–212. 10.3109/07388551.2015.1130683 26810104

[18] Romero FM, Marina M, Pieckenstain FL. Novel components of leaf bacterial communities of field-grown tomato plants and their potential for plant growth promotion and biocontrol of tomato diseases. Res Microbiol. 2016; 167: 222–233. 10.1016/j.resmic.2015.11.001 26654914

[19] de Lamo FJ, Constantin ME, Fresno DH, Boeren S, Rep M, Takken FLW. Xylem sap proteomics reveals distinct differences between *R* gene- and endophyte-mediated resistance against *Fusarium* wilt disease in tomato. Front Microbiol. 2018; 9: 2977 10.3389/fmicb.2018.02977 30564219 PMC6288350

[20] Marquez-Santacruz H, Hernandez-Leon R, Orozco-Mosqueda M, Velazquez-Sepulveda I, Santoyo G. Diversity of bacterial endophytes in roots of Mexican husk tomato plants (Physalisixocarpa) and their detection in the rhizosphere. Genet Mol Res. 2010; 9: 2372–2380. 10.4238/vol9-4gmr921 21157706

[21] Ottesen AR, González Peña A, White JR, Pettengill JB, Li C, Allard S, et al Baseline survey of the anatomical microbial ecology of an important food plant: *Solanum lycopersicum* (tomato). BMC Microbiol. 2013; 13: 114 10.1186/1471-2180-13-114 23705801 PMC3680157

[22] Romero FM, Marina M, Pieckenstain FL. The communities of tomato (*Solanum lycopersicum* L.) leaf endophytic bacteria, analyzed by 16S-ribosomal RNA gene pyrosequencing. FEMS Micorbiol Lett. 2015; 351: 187–194. 10.1111/1574-6968.12377 24417185

[23] Allard SM, Walsh CS, Wallis AE, Ottesen AR, Brown EW, Micallef SA. *Solanum lycopersicum* (tomato) hosts robust phyllosphere and rhizosphere bacterial communities when grown in soil amended with various organic and synthetic fertilizers. Sci Total Environ. 2016; 573: 555–563. 10.1016/j.scitotenv.2016.08.157 27580466

[24] Tian BY, Cao Y, Zhang KQ. Metagenomic insights into communities, functions of endophytes, and their associates with infection by root-knot nematode, *Meloidogyne incognita*, in tomato roots. Sci Rep. 2015; 5: 17087 10.1038/srep17087 26603211 PMC4658523

[25] Toju H, Okayasu K, Notaguchi M. Leaf-associated microbiomes of grafted tomato plants. Sci Rep. 2019; 9: 1787 10.1038/s41598-018-38344-2 30741982 PMC6370777

[26] Mori H, Maruyama F, Kato H, Toyoda A, Dozono A, Ohtsubo Y, et al Design and experimental application of a novel non-degenerate universal primer set that amplifies prokaryotic 16S rRNA genes with a low possibility to amplify eukaryotic rRNA genes. DNA Res. 2014; 21: 217–227. 10.1093/dnares/dst052 24277737 PMC3989492

[27] Tremblay J, Singh K, Fern A, Kirton ES, He S, Woyke T, et al Primer and platform effects on 16S rRNA tag sequencing. Front Microbiol. 2015; 6: 771 10.3389/fmicb.2015.00771 26300854 PMC4523815

[28] Coleman-Derr D, Desgarennes D, Fonseca-Garcia C, Gross S, Clingenpeel S, Woyke T, et al Plant compartment and biogeography affect microbiome composition in cultivated and native *Agave* species. New Phytol. 2016; 209: 798–811. 10.1111/nph.13697 26467257 PMC5057366

[29] Rezki S, Campion C, Iacomi-Vasilescu B, Preveaux A, Toualbia Y, Bonneau S, et al Differences in stability of seed-associated microbial assemblages in response to invasion by phytopathogenic microorganisms. Peer J. 2016; 4: e1923 10.7717/peerj.1923 27077013 PMC4830237

[30] Edgar RC, Haas BJ, Clemente JC, Quince C, Knight R. UCHIME improves sensitivity and speed of chimera detection. Bioinformatics 2011; 27: 2194–2200. 10.1093/bioinformatics/btr381 21700674 PMC3150044

[31] Bodenhausen N, Horton MW, Bergelson J. Bacterial communities associated with the leaves and the roots of *Arabidopsis thaliana*. PLoS ONE 2013; 8: e56329 10.1371/journal.pone.0056329 23457551 PMC3574144

[32] Amato KR, Yeoman CJ, Kent A, Righini N, Carbonero F, Estrada HR, et al Habitat degradation impacts black howler monkey (*Alouatta pigra*) gastrointestinal microbiomes. ISME J. 2013; 7: 1344–1353. 10.1038/ismej.2013.16 23486247 PMC3695285

[33] Schloss PD, Gevers D, Westcott SL. Reducing the effects of PCR amplification and sequencing artifacts on 16S rRNA-based studies. PLoS ONE 2011; 6: e27310 10.1371/journal.pone.0027310 22194782 PMC3237409

[34] Lozupone C, Knight R. UniFrac: a new phylogenetic method for comparing microbial communities. Appl Environ Microbiol. 2005; 71: 8228–8235. 10.1128/AEM.71.12.8228-8235.2005 16332807 PMC1317376

[35] Yashiro E, McManus PS. Effect of streptomycin treatment on bacterial community structure in the apple phyllosphere. PLoS ONE 2012; 7: e37131 10.1371/journal.pone.0037131 22629357 PMC3357425

[36] Delmotte N, Knief C, Chaffron S, Innerebner G, Roschitzki B, et al Community proteogenomics reveals insights into the physiology of phyllosphere bacteria. Proc Natl Acad Sci USA. 2009; 106: 16428–16433. 10.1073/pnas.0905240106 19805315 PMC2738620

[37] Knief C, Delmotte N, Chaffron S, Stark M, Inn erebner G, et al Metaproteogenomic analysis of microbial communities in the phyllosphere and rhizosphere of rice. ISME J. 2012; 6:1378–1390. 10.1038/ismej.2011.192 22189496 PMC3379629

[38] Lindow SE, Leveau JH. Phyllosphere microbiology. Curr Opin Biotechnol. 2002; 13: 238–243. 10.1016/s0958-1669(02)00313-0 12180099

[39] Rastogi G, Coaker GL, Leveau JHJ. New insights into the structure and function of phyllosphere microbiota through high-throughput molecular approaches. FEMS Microbiol Lett. 2013; 348: 1–10. 10.1111/1574-6968.12225 23895412

[40] Hardoim PR, van Overbeek LS, van Elsas JD. Properties of bacterial endophytes and their proposed role in plant growth. Trends Microbiol. 2008; 16: 463–471. 10.1016/j.tim.2008.07.008 18789693

[41] Andreote FD, Pereira E Silva MC. Microbial communities associated with plants: learning from nature to apply it in agriculture. Curr Opin Microbiol. 2017; 37: 29–34. 10.1016/j.mib.2017.03.011 28437663

[42] Czajkowski R, de Boer WJ, Velvis H, van der Wolf JM. Systemic colonization of potato plants by a soilborne, green fluorescent protein-tagged strain of Dickeya sp. biovar 3. Phytopathology 2010; 100: 134–142. 10.1094/PHYTO-100-2-0134 20055647

[43] Wang W, Zhai Y, Cao L, Tan H, Zhang R. Endophytic bacterial and fungal microbiota in sprouts, roots and stems of rice (*Oryza sativa* L.). Microbiol Res. 2016; 188–189: 1–8. 10.1016/j.micres.2016.04.009 27296957

[44] Gans J, Wolinsky M, Dunbar J. Computational improvements reveal great bacterial diversity and high metal toxicity in soil. Science 2005; 309:1387–1390. 10.1126/science.1112665 16123304

[45] Hardoim PR, Andreote FD, Reinhold-Hurek B, Sessitsch A, van Overbeek LS, van Elsas JD. Rice root-associated bacteria: insights into community structures across 10 cultivars. FEMS Microbiol Ecol. 2011; 77: 154–164. 10.1111/j.1574-6941.2011.01092.x 21426364 PMC4339037

[46] Tian XY, Zhang CS. Illumina-based analysis of endophytic and rhizosphere bacterial diversity of the coastal halophyte *Messerschmidia sibirica*. Front Microbiol. 2017; 8: 2288 10.3389/fmicb.2017.02288 29209296 PMC5701997

[47] Lee SA, Park J, Chu B, Kim JM, Joa JH, Sang MK, et al Comparative analysis of bacterial diversity in the rhizosphere of tomato by culture-dependent and -independent approaches. J Microbiol. 2016; 54: 823–831. 10.1007/s12275-016-6410-3 27888459

[48] Magnucka EG, Pietr SJ. Various effects of fluorescent bacteria of the genus *Pseudomonas* containing ACC deaminase on wheat seedling growth. Microbiol Res. 2015; 181: 112–119. 10.1016/j.micres.2015.04.005 25983132

[49] Rastogi G, Sbodio A, Tech JJ, Suslow TV, Coaker GL, Leveau JH. Leaf microbiota in an agroecosystem: spatiotemporal variation in bacterial community composition on field-grown lettuce. ISME J. 2012; 6:1812–1822. 10.1038/ismej.2012.32 22534606 PMC3446804

[50] Manter DK, Delgado JA, Holm DG, Stong RA. Pyrosequencing reveals a highly diverse and cultivar-specific bacterial endophyte community in potato roots. Microb Ecol. 2010; 60: 157–166. 10.1007/s00248-010-9658-x 20414647

[51] Lopez-Velasco G, Tydings HA, Boyer RR, Falkinham JO 3rd, Ponder MA. Characterization of interactions between *Escherichia coli* O157:H7 with epiphytic bacteria in vitro and on spinach leaf surfaces. Int J Food Microbiol. 2012; 153: 351–357. 10.1016/j.ijfoodmicro.2011.11.026 22177225

[52] Compant S, Kaplan H, Sessitsch A, Nowak J, Barka EA, Clement C. Endophytic colonization of *Vitis vinifera* L. by *Burkholderia phytofiimans* strain PsJN: from the rhizosphere to inflorescence tissues. FEMS Microbiol Ecol. 2008; 63: 84–93. 10.1111/j.1574-6941.2007.00410.x 18081592

[53] Zhao J, Xu Y, Ding Q, Huang X, Zhang Y, Zou Z, et al Association mapping of main tomato fruit sugars and organic acids. Front Plant Sci. 2016; 7: 1286 10.3389/fpls.2016.01286 27617019 PMC4999453

[54] Amari M, Arango LF, Gabriel V, Robert H, Morel S, Moulis C, et al Characterization of a novel dextransucrase from *Weissella confusa* isolated from sourdough. Appl Microbiol Biotechnol. 2013; 97: 5413–5422. 10.1007/s00253-012-4447-8 23053097

[55] Porter NT, Luis AS, Martens EC. Bacteroides thetaiotaomicron. Trends Microbiol. 2018; 26: 966–967. 10.1016/j.tim.2018.08.005 30193959

[56] Halpern M, Fridman S, Atamna-Ismaeel N, Izhaki I. *Rosenbergiella nectarea* gen. nov., sp. nov., in the family Enterobacteriaceae, isolated from floral nectar. Int J Syst Evol Microbiol. 2013; 63: 4259–4265. 10.1099/ijs.0.052217-0 23832968

