# Supplementary material for: Bacterial communities in the rhizosphere, phyllosphere and endosphere of tomato plants
Source: PLoS One. 2019 Nov 8;14(11):e0223847. doi: 10.1371/journal.pone.0223847 (PMC6839845; doi:10.1371/journal.pone.0223847)
Supplement: S1 Table — (DOCX) [file pone.0223847.s002.docx]

**Supporting Information**

**Table S1 Statistics of Illumina sequencing data of epiphytic and endophytic bacteria associated with tomato plants**

| Sample |  | Before subsampling | | | | After subsampling | |
| --- | --- | --- | --- | --- | --- | --- | --- |
|  |  | **Valid sequences** | **Trimmed sequences** | **Bacterial sequences** | **Bacterial OTUs^*^** | **Bacterial sequences** | **Bacterial OTUs^*^** |
| Root zone soil | R1 | 31 341 | 16 966 | 16 966 | 1 189 | 16 966 | 1 189 |
|  | R2 | 37 240 | 24 847 | 24 844 | 1 312 | 16 966 | 1 256 |
|  | R3 | 40 454 | 29 853 | 29 847 | 1 316 | 16 966 | 1 250 |
| Rhizosphere | R1 | 33 103 | 24 740 | 24 432 | 656 | 16 966 | 569 |
|  | R2 | 38 266 | 30 916 | 30 865 | 760 | 16 966 | 613 |
|  | R3 | 37 886 | 28 976 | 28 908 | 677 | 16 966 | 568 |
| Ep-Stem | R1 | 32 376 | 31 697 | 31 494 | 34 | 16 966 | 31 |
|  | R2 | 44 118 | 43 268 | 43 192 | 33 | 16 966 | 31 |
|  | R3 | 36 000 | 35 355 | 35 284 | 43 | 16 966 | 37 |
| Ep-Leaf | R1 | 37 273 | 36 062 | 35 632 | 89 | 16 966 | 74 |
|  | R2 | 30 133 | 29 232 | 28 866 | 95 | 16 966 | 80 |
|  | R3 | 31 822 | 31 075 | 30 648 | 76 | 16 966 | 75 |
| En-Root | R1 | 39 744 | 36 615 | 28 382 | 96 | 16 966 | 83 |
|  | R2 | 35 031 | 33 520 | 27 777 | 121 | 16 966 | 99 |
|  | R3 | 39 900 | 35 743 | 27 030 | 95 | 16 966 | 87 |
| En-Stem | R1 | 33 344 | 32 708 | 24 880 | 27 | 16 966 | 25 |
|  | R2 | 36 551 | 35 965 | 25 124 | 29 | 16 966 | 27 |
|  | R3 | 33 106 | 32 411 | 27 638 | 46 | 16 966 | 43 |
| En-Leaf | R1 | 32 351 | 30 366 | 25 149 | 23 | 16 966 | 23 |
|  | R2 | 42 798 | 39 446 | 26 125 | 35 | 16 966 | 33 |
|  | R3 | 41 461 | 38 934 | 21 617 | 25 | 16 966 | 23 |
| En-Pericarp | R1 | 30 474 | 29 241 | 22 986 | 22 | 16 966 | 22 |
|  | R2 | 40 383 | 38 658 | 30 095 | 22 | 16 966 | 18 |
|  | R3 | 37 769 | 36 410 | 28 491 | 31 | 16 966 | 31 |
| En-Placenta | R1 | 42 742 | 42 121 | 18 402 | 28 | 16 966 | 26 |
|  | R2 | 43 720 | 43 216 | 19 006 | 30 | 16 966 | 28 |
|  | R3 | 42 246 | 41 622 | 21 888 | 37 | 16 966 | 36 |
| En-Jelly | R1 | 50 843 | 50 173 | 17 112 | 23 | 16 966 | 23 |
|  | R2 | 52 624 | 52 151 | 21 135 | 22 | 16 966 | 21 |
|  | R3 | 52 434 | 51 866 | 20 769 | 32 | 16 966 | 30 |
| En-Seed | R1 | 56 808 | 56 114 | 17 523 | 30 | 16 966 | 28 |
|  | R2 | 54 713 | 53 957 | 18 486 | 34 | 16 966 | 30 |
|  | R3 | 54 868 | 54 225 | 17 321 | 31 | 16 966 | 30 |

^*^ OTUs were classified at 97% similarity.
