# Supplementary material for: Bacterial communities in the rhizosphere, phyllosphere and endosphere of tomato plants
Source: PLoS One. 2019 Nov 8;14(11):e0223847. doi: 10.1371/journal.pone.0223847 (PMC6839845; doi:10.1371/journal.pone.0223847)
Supplement: S1 Fig — (DOCX) [file pone.0223847.s003.docx]

**Supporting information**

**Supplemental Figure S1**


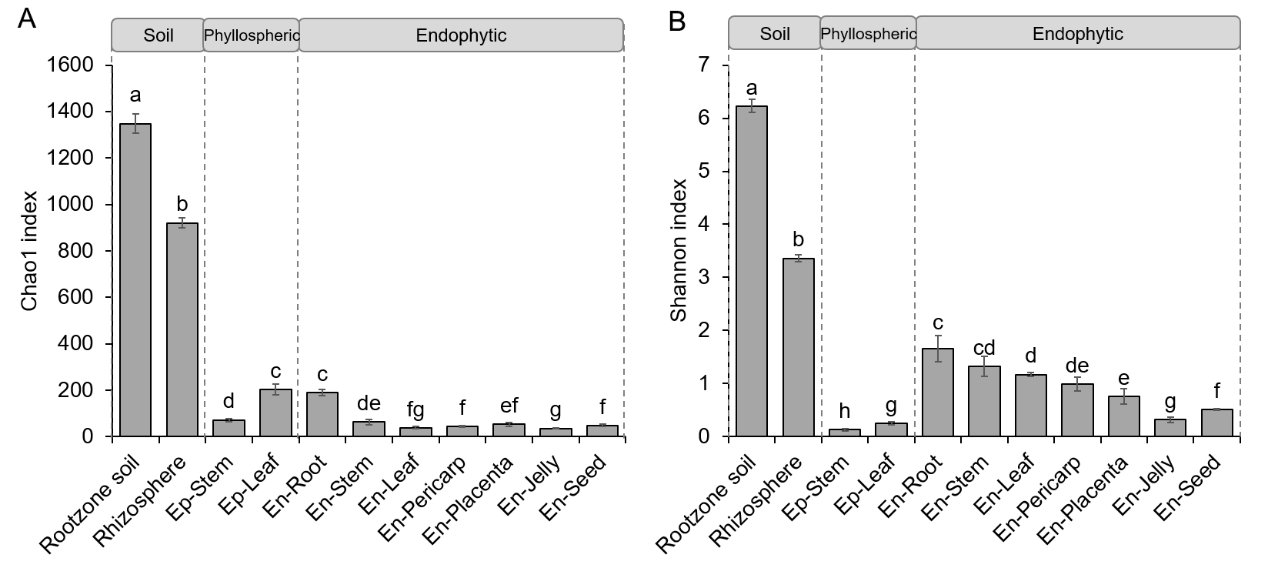


**Figure S1. Chao1 (A) and Shannon (B) indexes of bacterial communities from root zone soil, rhizosphere, phyllosphere and endosphere of tomato plants before subsampling.**
