# Supplementary material for: Bacterial communities in the rhizosphere, phyllosphere and endosphere of tomato plants
Source: PLoS One. 2019 Nov 8;14(11):e0223847. doi: 10.1371/journal.pone.0223847 (PMC6839845; doi:10.1371/journal.pone.0223847)
Supplement: S2 Fig — (DOCX) [file pone.0223847.s004.docx]

**Supporting information**

**Supplemental Figure S2**


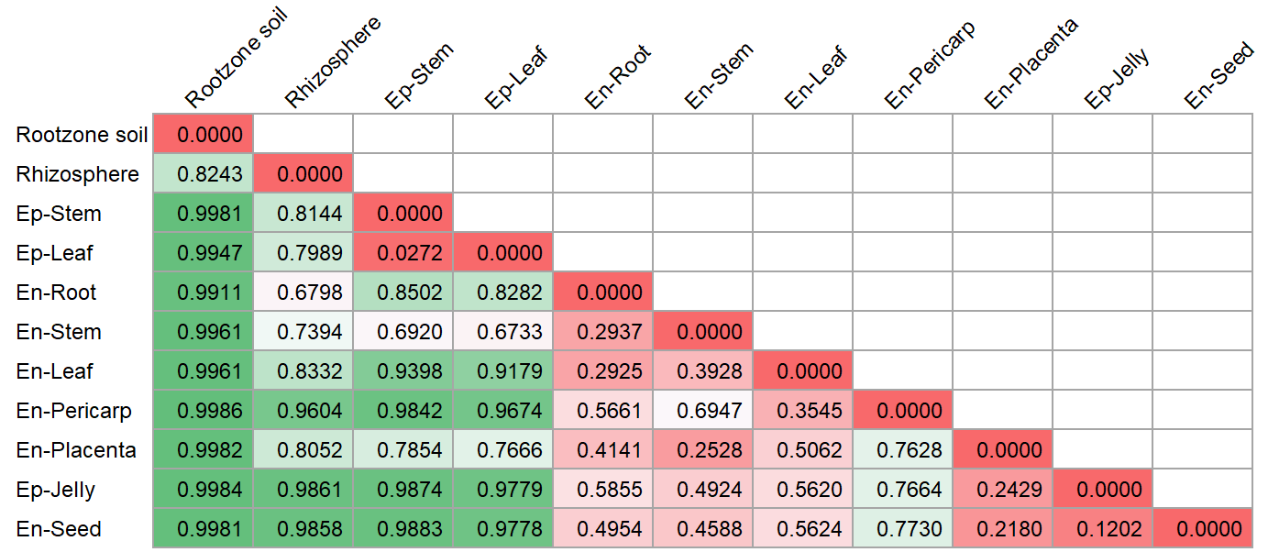


**S2 Fig. Pairwise Bray-Curtis dissimilarity indexes across the bacterial communities from the root zone soil, rhizosphere, phyllosphere and endosphere of tomato plants.**
